# A Survey of Routing Protocols in Wireless Body Sensor Networks

**DOI:** 10.3390/s140101322

**Published:** 2014-01-13

**Authors:** Javed Iqbal Bangash, Abdul Hanan Abdullah, Mohammad Hossein Anisi, Abdul Waheed Khan

**Affiliations:** Faculty of Computing, Universiti Teknologi Malaysia (UTM), 81310 Skudai Johor, Malaysia; E-Mails: ibjaved2@live.utm.my (J.I.B.); anisi@utm.my (M.H.A.); wkabdul2@live.utm.my (A.W.K.)

**Keywords:** Wireless Body Sensor Networks, Wireless Sensor Networks, routing protocols

## Abstract

Wireless Body Sensor Networks (WBSNs) constitute a subset of Wireless Sensor Networks (WSNs) responsible for monitoring vital sign-related data of patients and accordingly route this data towards a sink. In routing sensed data towards sinks, WBSNs face some of the same routing challenges as general WSNs, but the unique requirements of WBSNs impose some more constraints that need to be addressed by the routing mechanisms. This paper identifies various issues and challenges in pursuit of effective routing in WBSNs. Furthermore, it provides a detailed literature review of the various existing routing protocols used in the WBSN domain by discussing their strengths and weaknesses.

## 1. Introduction

A Wireless Sensor Network (WSN) refers to a distributed network, consisting of dispersed and autonomous sensing stations. Each sensing station—also known as a sensor node—consists of a microcomputer (computing component), transceiver (communication component), a power source (normally a battery), and some sensor(s) depending upon the application area. Some smart sensors are equipped with an actuator [1]—an electro-mechanical device used to control different components of the system. These nodes self-organize themselves upon deployment and form a network, which is typically comprised of several to thousands of such sensor nodes. Upon forming the network, sensor nodes sense, measure and gather information from the surrounding environment for some activity and report the sensed data to a special station called base station or sink in a multi-hop fashion.

WSNs can be deployed on a large or small scale (depending on the requirements and applications) to detect and collect the required information from the surrounding environment. They can be deployed either in a pre-planned manner or on random basis. WSNs have the ability to change people's lifestyle [2]. They can be used for different applications [3], such as environmental monitoring and seismic sensing [4], natural disaster relief [5], bio-medical health monitoring [6–9], and military target tracking, national defense and surveillance [10,11].

According to Department of Economic and Social Affairs of United Nations Secretariat [12], by year 2025, people of 65+ age will be account for about 15% of the overall worldwide population, which will be nearly 761 millions [13]. In developed countries the elderly population will be high, *i.e.*, 20% [14] as compared to developing and under-developed countries. Since the elderly aged people are more vulnerable to different health issues and diseases, they require frequent medical check-up, which results in high healthcare costs [15,16]. These statistics demand major changes towards proactive management of these issues by focusing on the prevention and early detection and treatment of different diseases [17].

Wireless Body Sensor Networks (WBSNs) are a subset of wireless sensor networks, which can offer this paradigm shift and can be used for early detection of the different diseases. They can collect and analyze the vital sign-related data of patients by deploying different types of bio-medical sensors (for example: body temperature, heartbeat, blood pressure, electrocardiogram (ECG), electro encephalogram (EEG), *etc.* sensors) for a long period of time, thus reducing the healthcare costs. The bio-medical sensor node can either be suitably placed on the body or implanted inside the body. These bio-medical sensor nodes send the sensed information to a coordinator (base station), located on or near the body. The coordinator (base station) is responsible for forwarding the collected information to the sink node. The sink node will send the received data to the health care center or any other destination.

In this paper a comprehensive study of the existing data routing approaches proposed during the last decade is provided, along with a critical analysis of each protocol. Section 2 covers the general architecture of the wireless body sensor networks, while in Section 3 the different routing issues and challenges of WBSNs are discussed. Based on the nature and structure of the existing routing protocols, they are classified into different classes and discussed in Section 4. Finally, this paper is concluded in Section 5.

## 2. Architecture of Wireless Body Sensor Networks

**Figure 1.**
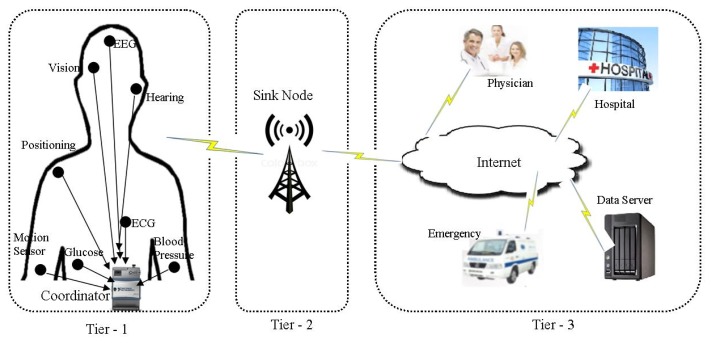
Architecture of Wireless Body Sensor Networks.

The architecture of WBSNs can divided into following three different tiers [18], as shown in Figure 1:

*Tier 1*—*Intra-WBSN*: In Intra-WBSN, the on-body and/or implanted bio-medical sensor nodes send the sensed data to the coordinator or base station.
*Tier 2*—*Inter-WBSNs*: In Inter-WBSN, coordinators or base stations send the received data to the sink(s) after required data processing and data aggregation.
*Tier 3*—*Extra-WBSN*: In this tier the sink(s) send the collected data to the remote medical center and/or any other destination via regular infrastructure such as internet.

## 3. Routing Issues and Challenges in WBSNs

Design and development of efficient routing protocols for WBSNs is a challenging job due to their unique requirements and specific characteristics [18]. In the following sections, we discuss the routing issues and challenges of WBSNs.

### 3.1. Network Topology

Network topology describes the logical way in which the different communicating devices communicate with each other. Efficient routing protocol development requires a proper network topology as it effects the overall performance of the communication system [19]. Proper network topology is very important for WBSNs because of the energy constraint, body postural movements, heterogeneous nature of the sensors and short transmission range. Some researchers use single hop communication, where each node communicates directly with the destination, while others use cluster based multi-hop routing, and are discussed later in this paper.

### 3.2. Topological Partitioning

The network topology of WBSNs often faces the problem of disconnection or partitioning because of body postural movements and short range transmissions. Different researchers have tried to solve the problem of disconnection and partitioning in different ways. For example, the authors of [20] use Line-of-Sight (LoS) and None-Line-of-Sight (NLoS) communication, while the authors of [21–23] use store-and-forward routing to solve this problem. Therefore, the proposed routing protocols should take care of the different topological changes.

### 3.3. Energy Efficiency

Energy efficiency covers both the local energy consumption of nodes and the overall network lifetime. For implanted bio-medical sensors, it is not possible to replace the power source, while for wearable bio-medical sensors replacing the batteries might lead to discomfort of patients. Therefore, both energy consumption and network lifetime are major challenges in wireless body sensor networks. Communication among the sensor nodes consumes more energy as compared to sensing and processing [24]. Any proposed algorithm should be able to use different paths and/or nodes to send the data instead of depending on a single path and/or node preventing the consumption of total energy of that specific node(s). In [22], the authors define the network life as the time from which the network starts till the time when the first node of the network expires. The network life is very much important in WBSNs because of energy constraints and the impossibility of replacing the energy source for implanted sensors.

### 3.4. Limited Resources

Along with limited energy source, WBSNs also have short Radio Frequency (RF) transmission range, poor computation capabilities, limited storage capacity, as well as low bandwidth—which may keep on changing due to noise and other interferences [19]. Researchers must be aware of the limited resources when designing routing protocols for WBSNs.

### 3.5. Quality of Service (QoS)

In WBSNs different types of data require different quality of services as it deals with vital signs of the human body. The authors in [25,26] have classified the patient data into critical data (like EEG, ECG *etc.*), delay sensitive data (for example video streaming), reliability-sensitive data (like vital signals monitoring respiration monitor, and PH monitor) and ordinary data (for example temperature, heartbeat, *etc.*). The other data-centric applications of WSNs also cannot tolerate latency and/or any loss of packets [27]. The proposed protocols need to be aware of the different types of quality of service required for different types of patients' vital sign- related data.

### 3.6. Radiation Absorption and Overheating

The two sources of temperature rise of a node are antenna radiation absorption and power consumption of node circuitry [28], which will affect the heat sensitive organs of the human body [28] and may damage some tissues [29]. Researchers should carefully develop the routing protocols for WBSNs to keep human tissues safe from any overheating caused by radiation absorption and operation of the implanted bio-medical sensor nodes.

### 3.7. Heterogeneous Environment

Different types of sensor nodes are required to sense and monitor the different health parameters of human beings, which may also differ in computation, storage capabilities and energy consumption [19]. Thus the heterogeneous nature of WBSNs also imposes some more challenges.

### 3.8. Path Loss

Path loss or path attenuation is a measure of the decline in power density of an electromagnetic wave as it propagates through the wireless medium. It is the ratio of the power of transmitted to received signals [30]. The wireless communication between the implanted sensor nodes is through the human body, where the path loss exponent varies from four to seven [31], which is very high as compared to the free space, where it is two. The researcher must consider the path loss while designing routing protocols for wireless body sensor networks.

### 3.9. Security and Privacy

Like other applications of WSNs, security and privacy are among the basic requirements of WBSNs. It is impossible to apply the conventional techniques of security and privacy because of the low energy availability, limited resources and other constraints [3]. Researchers should take care of the privacy and security of the patient's data while designing routing protocols for WBSNs.

## 4. Classification of Routing Protocols

**Figure 2.**
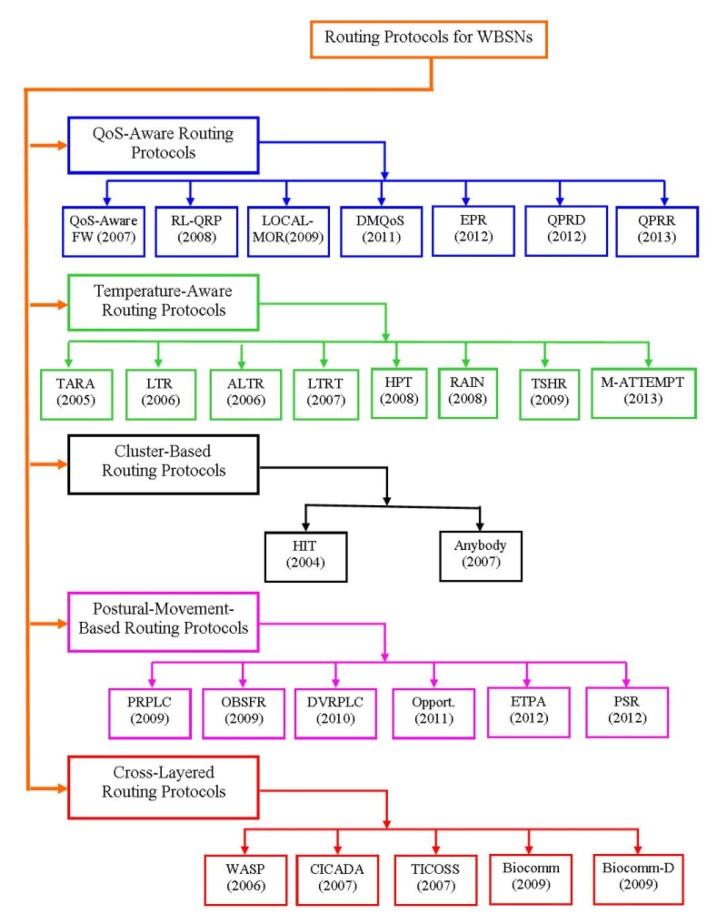
Classification of routing protocols for WBSNs.

During last decade, researchers have proposed different types of routing protocols. In this section, we provide a general overview of the existing routing algorithms, which may be classified as QoS-aware routing protocols, temperature-aware routing protocols, cluster-based routing protocols, postural-movement-based routing protocols and cross-layered routing protocols, as shown in Figure 2.

### 4.1. QoS-Aware Routing Protocols

The QoS-aware routing protocols are modular-based protocols and use different modules for different types of QoS metrics. The design of these protocols is a challenging job, due to the complexity of considering different modules for different QoS metrics and coordination between these modules. In the following sections different QoS-aware routing protocols for WBSNs are discussed.

#### 4.1.1. Routing Service Framework

**Figure 3.**
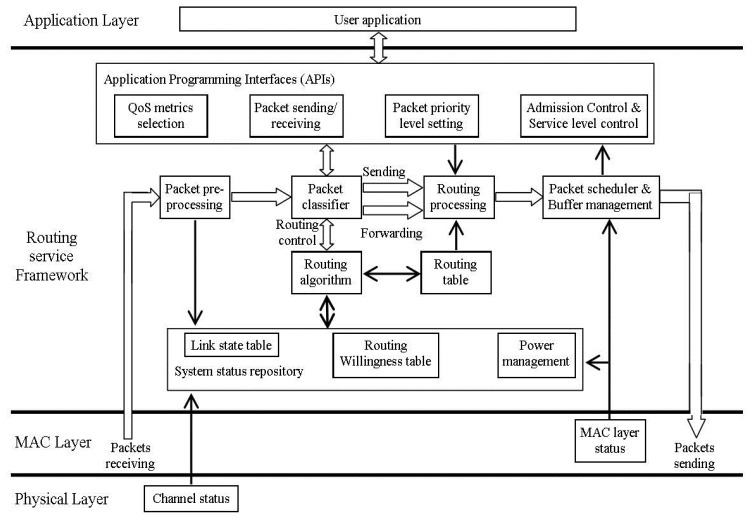
Architecture of QoS-aware routing service framework [32].

A cross-layered modular based QoS-aware routing service framework proposed in [32] aims to provide priority-based routing services and user specific QoS support. The QoS metrics used to determine the routes are: user specific QoS requirements, wireless channel status, priority level of the data packets and willingness of the sensor nodes to behave as a router. The main functions of this framework are: QoS-aware route establishment and maintenance, prioritized packet routing, Application Programming Interfaces (APIs), feedback on network condition to the user application(s) and finally adaptive network traffic balancing. As shown in Figure 3, redrawn from [32], the architecture of this routing service framework has four modules: Application Programming Interfaces (APIs) module, routing service module, packet queuing and schedule module and system information repository module.

The APIs module of [32] acts as an interface between the user application and the routing service module. The four sub-modules of the APIs module are: QoS metrics selection, packet sending/receiving, packet priority level setting and admission control and service level control. The QoS metrics selection sub-module includes end-to-end delay, delivery ratio and power consumption. The packet sending/receiving sub-module is responsible for receiving the sensed data from the user application and sending it to sink node or any other node. The packet priority level setting sub-module is responsible for setting the priority level of the received data packets. Finally, the admission control and service level sub-module control returns feedback on the network conditions to the user application. The second module (routing service module) is responsible for constructing and maintaining the routing table with the help of the receiving neighbor's status information. All data packets, including both the control and data packets, are categorized into eight different priority levels, where the control packets have more priority as compared to the data packets. The node's buffer will reach a pre-assigned threshold value if the sensor node is not able to access the wireless channel due to network congestion. In such cases the packet queuing and scheduling module will inform the user application to reduce service level and willingness level to be a router. The System Information Repository module maintains two tables: link state table and willingness table. The link state table provides the link state of each node, including link quality, end-to-end delay, communication bandwidth, and average packet delivery ratio, while the willingness table contains the information for each node to behave as a router.

#### 4.1.2. Reinforcement Learning based Routing Protocol with QoS Support (RL-QRP)

In [33], the authors proposed a reinforcement learning-based routing protocol with QoS support, using geographic information and a distributed Q-learning algorithm where the optimal routes can be found through experiences and rewards. The tiny bio-medical sensor nodes, implanted inside the body or attached with the body, forward the sensed data to sink nodes deployed at fixed positions. After collecting the data packets from the bio-medical sensor nodes, the sink node(s) forward them to the medical server for further real-time monitoring and diagnosis.

In this scheme the packet delivery ratio and end-to-end delay are the main QoS metrics. In the Q-learning algorithm, each sensor node receives a reward, either positive or negative, after forwarding a data packet to its neighbor. The reward along with the expected future reward, updates the Q-value of the sensor node, which will be used for the future decisions. Sensor nodes exchange the Q-values with its one-hop neighbors to learn about their optimal routes. The sensor nodes can use the neighbor sensor nodes Q-value information to predict the expected future reward. Each sensor node considers its Q-values list as its routing table.

The authors of this scheme use Random Waypoint Mobility Model (RWMM) for the mobile sensor nodes, where the sensor nodes can only move to the chosen random destinations and will stay there for a predefined time. RL-QRP uses the neighbor nodes' Q-values and geographic information to find out the optimal routes while energy which is one of the major constraints of wireless sensor networks, is not considered at all.

#### 4.1.3. New QoS and Geographic Routing (LOCALMOR)

A distributed QoS aware module-based protocol is proposed in [25], to help the system to meet different QoS requirements based on the nature of data (energy efficiency, reliability and latency). The proposed mechanism divides the patient's data into: Regular Traffic, Reliability-Sensitive Traffic, Delay-Sensitive Traffic, and Critical Traffic. The coordinator, which they called a body sensor mote collects the raw data from the bio-medical sensor nodes and after required data processing, and data aggregation, sends it to the sink node(s). Each (fixed) sink node may cover more than one patient (fixed and/or mobile). The proposed protocol has two kinds of sink nodes for every patient: Primary Sink and Secondary Sink and each sink receive a separate copy of each message.

**Figure 4.**
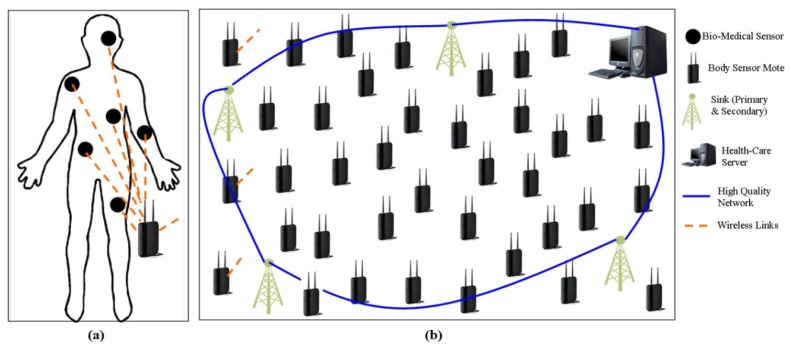
System architecture of LOCALMOR, (**a**) On-body network, (**b**) In-hospital network.

In their scheme, they use four different modules: power efficiency module, reliability-sensitive module, delay-sensitive module, and neighbor manager module. The power efficiency module is responsible for the regular traffic data packets and may be used by other modules to optimize the data-related metrics. Power efficiency can be achieved by considering both the transmission power and residual energy using Min-Max Approach discussed in [34]. The reliability drawn data packets use the reliability-sensitive module to achieve the required reliability by sending a copy of each data packet to both the primary and secondary sinks. The delay-sensitive module is used to route the latency sensitive data packets by using Pocket Velocity Approach given in [35]. The neighbor manager module is responsible to send/receive the Hello packets and update neighbors' information. The system architecture of the proposed protocol is shown in Figure 4.

#### 4.1.4. Data-Centric Multi-Objectives QoS-Aware Routing (DMQoS)

**Figure 5.**
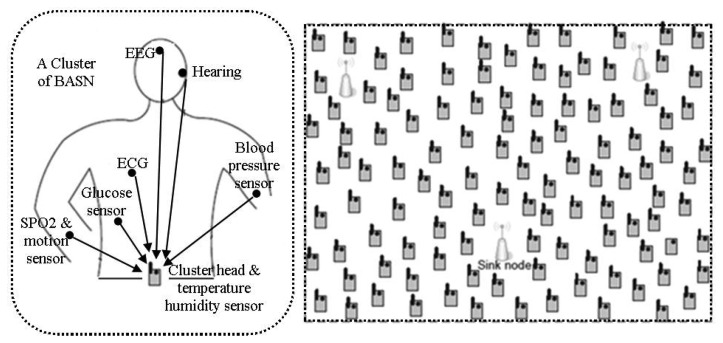
Network architecture for DMQoS [26].

DMQoS proposed in [26] is a module-based multi-objective QoS-aware routing protocol that focuses on meeting the QoS requirements for different categories of the generated data. In DMQoS, the data packets are divided into four classes: Ordinary Data Packets (ODs), Reliability-Driven Data Packets (RPs), Delay-Driven Data Packets (DPs) and Critical Data Packets (CPs). The bio-medical sensor nodes send the sensed data towards the coordinator, which they called as body sensor mote in raw form. The body sensor mote is a central node acting as a cluster head and having less constraints in terms of energy and computation capability as compared to bio-medical sensor nodes. The network architecture of the DMQoS is shown in Figure 5, redrawn from [26]. After the required data processing and aggregation, the body sensor mote forwards the data towards the sink in multi-hop fashion using other body sensor motes.

**Figure 6.**
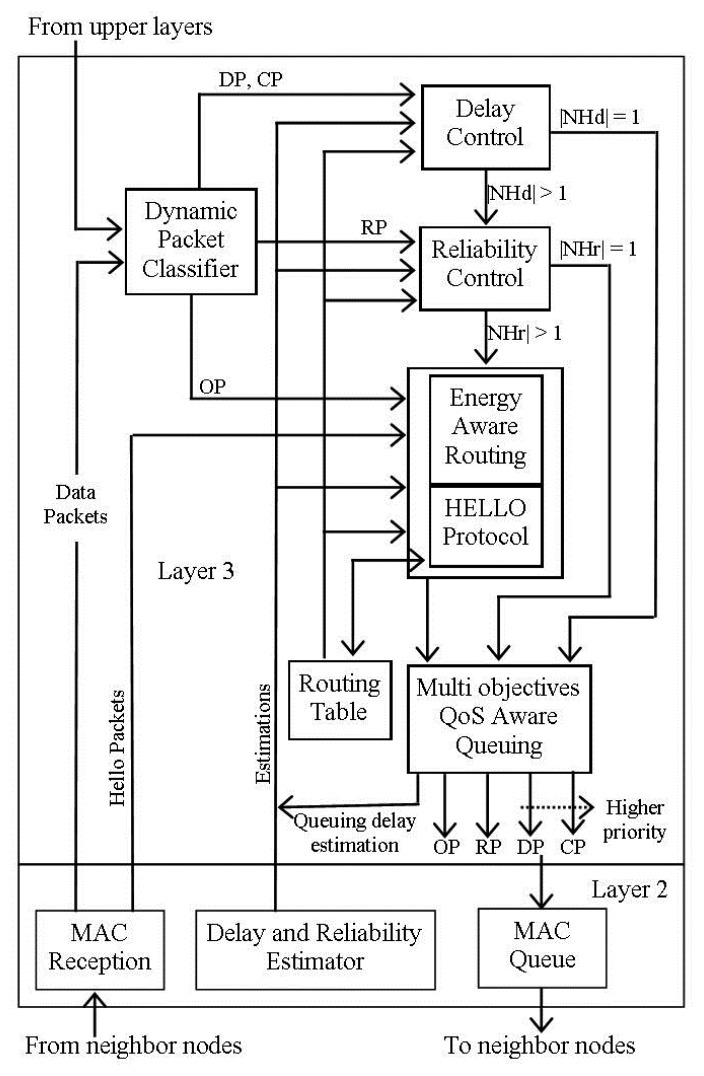
Routing architecture for DMQoS [26].

The routing architecture of DMQoS [26] consists of five modules: dynamic packet classifier, energy-aware geographic forwarding module, reliability control module, delay control module, and multi-objectives QoS aware queuing module, as shown in Figure 6, redrawn from [26]. The dynamic packet classifier receives the data packets from the neighbor node or the upper layers then classifies them into one of the four aforementioned categories, and forwards them to their respective module on a First-Come-First-Serve (FCFS) basis. The energy-aware geographic forwarding module decides the next hop node with least distance and comparatively high residual energy using multi-objective Lexicographic Optimization (LO) discussed in [36]. The reliability control module determines the next hop with highest reliability, while the delay control module finds the next hop having least delay. The QoS aware queuing module is responsible for forwarding the received data packet to one of the four queues based on the assigned priorities, as shown in Figure 6, redrawn from [26]. The use of the multi-objective LO approach to manage the tradeoff between the geographic information and residual energy ensures a homogenous energy consumption rate for all nodes.

#### 4.1.5. Energy-Aware Peering Routing (EPR)

**Figure 7.**
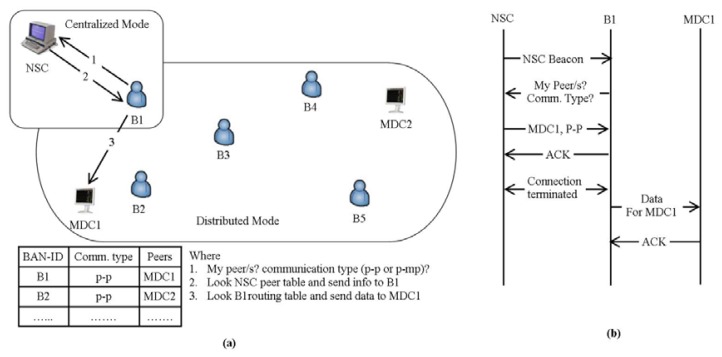
(**a**) EPR framework, (**b**) Timing diagram [37].

In [37] the authors presented an Energy-aware Peering Routing protocol aimed at reducing the network traffic and the energy consumption, based on both centralized and distributed approaches. It is designed to display the patients' real-time data inside a hospital. In this scheme, they have used three types of communication devices: Type 1—Nursing Station Coordinator (NSC), Type 2—Medical Display Coordinator (MDC), and Type 3—Body Area Network Coordinator (BANC). NCS is a centralized device with continuous power supply, which keeps the peering and type of communication information of all BANCs. MDCs are display devices with replaceable power supplies while the BANCs with limited energy, are responsible for collecting the data from the tiny bio-medical sensor nodes and forward it towards the corresponding MDC(s) after required processing. Initially, the BANC will try to access the NSC to get the peering and communication type (p-p or p-mp) information of MDC(s). After getting the required information, the BANC will discover the corresponding MDC(s) and display the data, as shown in Figure 7, redrawn from [37]. The energy efficiency is achieved by controlling the broadcasting mechanism of the hello packets. At the same time the selection of next hop node is based on the aforementioned device types, geographic information and residual energy of the neighbor.

#### 4.1.6. QoS-Aware Peering Routing for Delay-Sensitive Data (QPRD)

**Figure 8.**
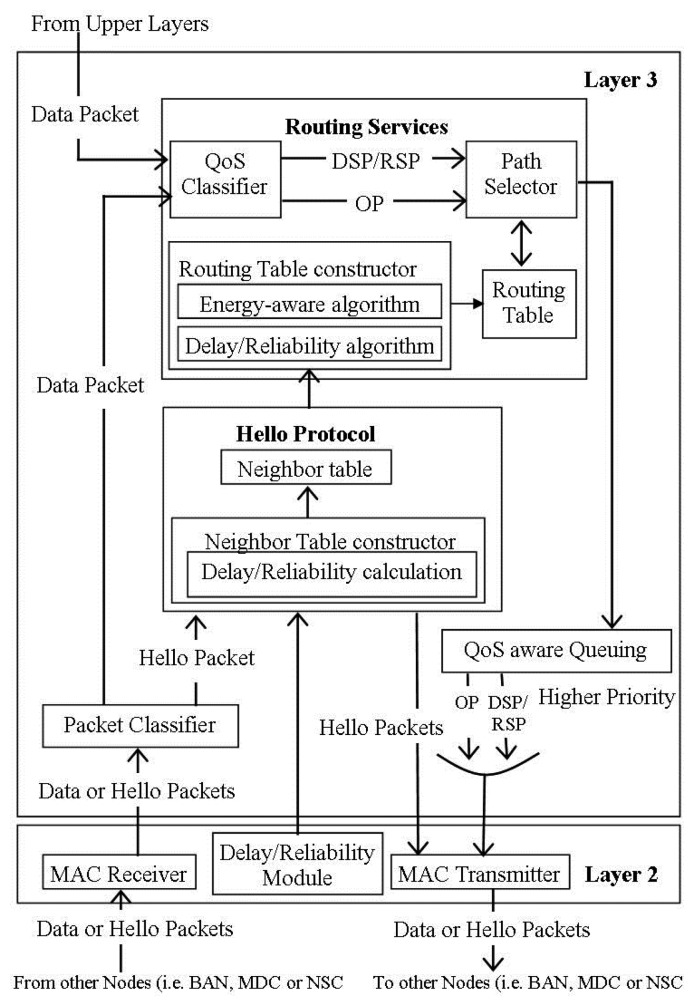
Routing architecture for QPRD and QPRR [39].

In [38] the authors proposed QPRD, which intends to improve the EPR discussed in [37] by classifying the patients' data packets into two categories: Ordinary Packets (OP) and Delay Sensitive Packets (DSP). It uses the same framework used by EPR [37]. As shown in Figure 8, redrawn from [39], the routing architecture of the QPRD is divided into seven modules: MAC receiver, delay module, packet classifier, hello protocol module, routing service module, QoS-aware queuing module, and MAC transmitter. The data packets from the other nodes are received by the MAC receiver module, while their classification as hello packets and data packets is done at the packet classifier module. The delay module monitors the different types of delays and forwards the results to the network layer to find out the node delay. The hello protocol module is responsible for sending/receiving the hello packets. The routing service module receives the data packets from the upper layers and packet classifier, categorizes them as ordinary packets or delay sensitive packets, and chooses the best path for each category. QoS-aware queuing module forwards the received data packets to their corresponding queue while the MAC transmitter module store the received data and hello packets in a queue on a First-Come-First-Serve (FCFS) manner and transmits them using the CSMA/CA approach.

#### 4.1.7. QoS-Aware Peering Routing for Reliability-Sensitive Data (QPRR)

QPRR is another QoS-aware peering routing protocol, proposed by Khan *et al.* in [39] designed to improve the EPR [37] by categorizing the patients' data into Ordinary Packets (OP) and Reliability Sensitive Packets (RSP). The routing architecture of this scheme is same as that of QPRD [38] shown in Figure 8, redrawn from [39] with a minor change. The delay module is replaced by a reliability module, which is responsible for monitoring and calculating the link reliability between any two nodes.

#### 4.1.8. Comparative Study of QoS-Aware Routing Protocols

Recently, different energy efficient and QoS-aware routing protocols have been proposed for wireless sensor networks [40–43], wireless multimedia sensor networks [44–46] and MANETs [47,48], which cannot be directly used for WBSNs because of their unique constraints. However, they can be incorporated after customization according to the dynamics of WBSNs. Furthermore, the QoS with dynamic wireless condition make the job more challenging [49]. Based on the literature review of QoS-aware routing protocols, which are discussed above, Table 1 provides a comparison in terms of network size, network throughput, mobility, delay, packet delivery ratio, and energy consumption with other state-of-the-art schemes.

The Routing Service Framework [32] aims to provide user specific QoS support uses a small scale network of 20 nodes and performs well in terms of reliability and latency, but it does not consider the energy consumption which is one of the major constraints of wireless sensor networks. Furthermore, it incurs more communication and computation overhead due to control packets. RL-QRP [33] is QoS-aware routing protocol based on geographic information and distributed Q-learning algorithm. As compared to QoS-AODV discussed in [50], RL-QRP performance is bad at the start due to learning process but becoming better with the passage of time. Like Routing Service Framework, it also uses small scale network of 20 nodes. The packet delivery ratio decreases as the mobility level increase and the average end-to-end delay increases with the increase in network throughput. Furthermore, this scheme does not consider the energy consumption.

LOCALMOR [25] is a modular approach that divides that patient's vital information into four categories. It performs well in terms of end-to-end delay, packet delivery ratio, and packet received within deadline (packet delivery delay) as compared to other state-of-the-art schemes. However, all data packets are blindly disseminated towards both the primary and secondary sinks. The network traffic increases due to sending too many duplicate data packets. To solve the issue of two sinks, each coordinator, which they called as body sensor mote, of DMQoS [26] sends the data packets towards a single sink. The different modules of DMQoS perform better to reduce latency, improve reliability, and decrease operational energy overloads as compared to other state-of-the-art approaches given in [25,51,52]. However, its performance decreases due to increase in the network throughput. Next, the use of the LO technique to optimize the trade-off between geographic information and residual energy is not an efficient way, because it ignores the less important function if the more important result in a unique solution. We argue that the next-hop selection may not optimal in terms of residual energy, as energy consideration is not being made if the geographic information results in a unique solution. Furthermore, in localized hop-by-hop approach the source node depends only on a single node's delay and/or reliability information. DMQoS uses large scale networks.

EPR [37] is the first peer-routing protocol based on both centralized and distributed approaches. It performance is better to reduce the energy consumption and network traffic as compared to DMQoS. QPRD [38] aims to improve EPR by categorizing the patient's vital signs information into ordinary packets and delay sensitive packets. It performs well to decrease the packet delivery delay as compared to DMQoS at high network throughput. Another QoS-aware routing protocol to improve EPR is QPRR [39], which classifies the patients' data packets into ordinary packets and reliability sensitive packets. It improves the packet delivery ratio at high network throughput than DMQoS. All the three protocols are designed to display the patient's data inside a hospital context. As compared to DMQoS, they use very small network size. Next, these protocols are suitable only for in-hospital environments. Furthermore, if the NSC is not accessible then the BANC will try to connect with nearest MDC, where the privacy of the patients and the communication control may be at risk.

By comparing the QoS-aware routing protocols in terms of energy efficiency, packet delivery delay and reliable data deliver, we conclude the following: in terms of energy consumption, EPR [37], QPRD [38] and QPRR [39] perform better as compared to others and all of them use the same module to reduce the energy consumption. On the other hand, both Routing Service Framework [32] and RL-QRP [33] do not consider the energy consumption. For large scale networks and with very low network throughput, DMQoS [26] performs better to reduce delay for delay sensitive data and improve reliability for reliability sensitive data. For small scale networks and with high network throughput, QPRD [38] results into less packet delivery delay while QPRR [39] improves the reliability of data delivery.

### 4.2. Temperature-Aware Routing Protocols

In wireless body sensor networks, some bio-medical sensors may be implanted inside the human body. Electric and magnetic fields are generated due to the radio signals used in wireless communication, which will result in temperature rise because of antenna radiation absorption, and power consumption of the node's circuitry [28]. This will affect temperature sensitive organs of human body by reducing the blood flow [28], growth of certain tissues, enzymatic reactions and might damage the surrounding tissues if in place for long periods of time [29]. Specific Absorption Rate (SAR) is the measure of the rate at which radiation energy is absorbed by the tissue per unit weight and is given in [28] as:

(1) $$\text{SAR}=\frac{\sigma {\left|E\right|}^{2}}{\rho }(W/Kg)$$

where ‘*σ*’ is electrical conductivity of tissue, ‘*E*’ is induced electric field by radiation and ‘*ρ*’ is density of tissue. During last decade, different researchers and engineers have tried to solve the problem of temperature rise and overheating. The aim of all temperature-aware routing protocols for WBSNs is to reduce the temperature rise of the implanted bio-medical sensor nodes. In the following sections, we have discussed the temperature-aware routing protocols for WBSNs proposed during last decade.

#### 4.2.1. Thermal-Aware Routing Algorithm (TARA)

**Figure 9.**
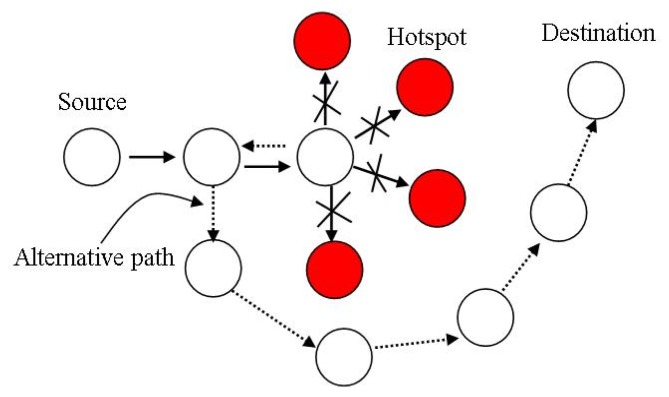
An example of TARA [15].

In [28], Tang *et al.*, proposed a thermal-aware routing algorithm that aims to reduce the possibility of overheating in implanted sensor networks. The implanted sensor network is a thermally sensitive application environment. The authors of [28] assumed that there is no sensor inside the bio-medical sensor nodes to measure the temperature of the node, because of the size and simplicity of bio-medical sensor nodes. In the setup phase each node observes the activities of its neighbor nodes, counts the received/transmitted packets and then evaluates their communication radiations and power consumption. Based on these observations each node estimates the changes in the temperature of its neighbors. Hotspots are the nodes having temperatures beyond the threshold value. In the routing phase the data packets whose destination is not the hotspot nodes are routed through nodes other than the hotspot nodes using withdrawal strategy. In the withdrawal strategy the nodes having hotspots as neighbor nodes send back the data packets to the sender node to find alternative routes to forward the data packet, as shown in Figure 9, redrawn from [15]. A data packet having a hotspot node as destination node, is buffered until the temperature of the destination node drops to a certain value.

In the TARA system model [28], bio-medical sensor nodes are physically implanted and the dielectric and perfusion properties of the tissues are assumed to be known. The implanted bio-medical sensors continuously sense the data and send it to the gateway which is responsible for aggregating the data and sending to the base station located outside the body. This algorithm calculates the Specific Absorption Rate (SAR) and temperature of each node using Finite Difference Time Domain (FDTD) discussed in [53] and Pennes Bioheat Equation given in [54].

#### 4.2.2. Least Temperature Rise (LTR)

**Figure 10.**
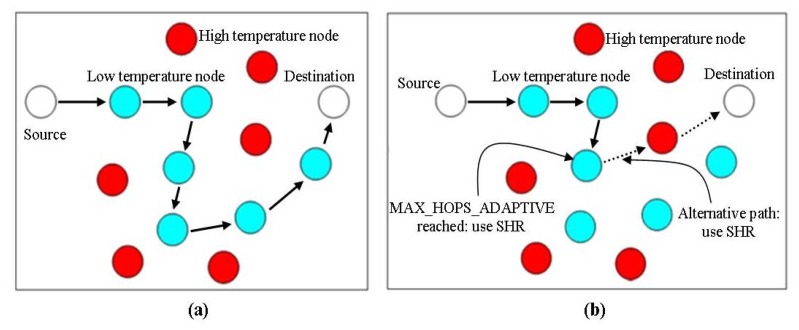
Example of (**a**) LTR, (**b**) ALTR [15].

A thermal-aware routing protocol named least temperature rise is presented in [55], to reduce the amount of heat produced in bio-medical sensor networks, power consumption and end-to-end delay. The researchers assume that each node knows the temperature information of its neighbor nodes by observing their communication activities. This scheme selects that node as next-hop node which has the least temperature until the destination is reached. Unlike TARA, which buffers the data packet if the destination is a hotspot node, it directly forwards the packet to the destination node. A hop-count is associated with each packet and is incremented by one each time a node forwards it. A packet is dropped if it exceeds the threshold value (MAX_HOPS), which depends on the diameter of the network. In addition to the hop-count, each packet also keeps record of nodes through which it passed to avoid looping. Figure 10a, redrawn from [15] shows an example of how LTR works.

#### 4.2.3. Adaptive Least Temperature Rise (ALTR)

The adaptive least temperature routing protocol (ALTR), proposed in [55], is designed to improve the LTR protocol. ALTR works in the same manner as LTR with a minor modification of replacing MAX_HOP by MAX_HOP_ADAPTIVE. In LTR if the hop-count of any packet exceeds the threshold value of MAX_HOP then it is discarded, however in ALTR it is not the same. If the hop-count of any packet exceeds the threshold value of MAX_HOP_ADAPTIVE, then a Shortest Hop Algorithm (SHA) is used to forward the packet towards its destination instead of dropping the packet. A sample of how ALTR works is shown in Figure 10b, redrawn from [15].

#### 4.2.4. Least Total Route Temperature (LTRT)

In [56], Takahashi *et al.*, proposed a thermal aware routing protocol named least total route temperature, designed to find the routes with minimum temperature as well as to address the problem of redundant hops. This protocol selects routes with total least temperature from the source node to the destination node and also saves the bandwidth of the network by decreasing the hop count. Single Source Shortest Path (SSSP) algorithms from graph theory (for example: Dijkstra's Algorithm) are used to calculate the route with total least temperature and then use these routes for data communication. This scheme monitors the communication activities of the neighbor nodes to collect their temperature information and accordingly assign the estimated temperature of each node as weight of that node. The objective of LTRT can be achieved by the following steps.

- Transfer the node's weight to outgoing edges weight of that node which results in a weight graph to calculate all possible routes from source node to the destination node.
- Apply Single Source Shortest Path (SSSP) algorithm to the weights graph of the nodes in order to figure out the routes with total least temperature from the source node to the destination node.
- Periodically update the routes to avoid the excessive temperature rise of the nodes.

**Figure 11.**
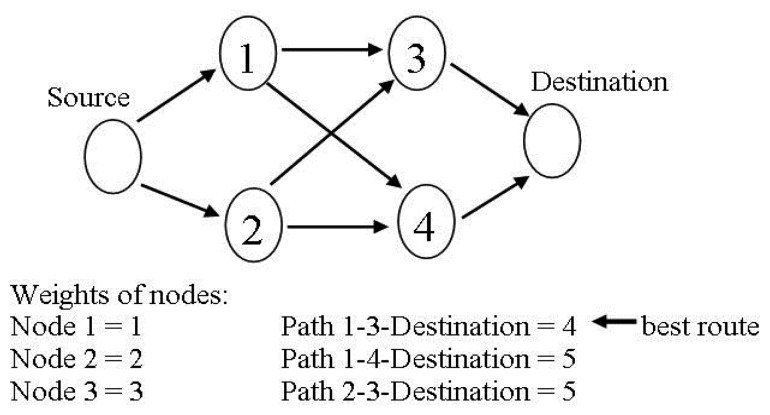
An example of LTRT [15].

Figure 11, redrawn from [15], show any example which explains that how LRTR works.

#### 4.2.5. Hotspot Preventing Routing (HPR)

The Hotspot Preventing Routing is a thermal-aware routing protocol for delay sensitive data, presented in [29]. The aim of this algorithm is to prevent the formation of the hotspots in the network as well as to minimize the average end-to-end delay. The two phases of HPR are: setup phase and routing phase. In the setup phase, all nodes exchange the information of the shortest path and initial temperature. Based on this information, each node constructs its own routing table. While in the second phase, *i.e.*, routing phase, each node considers the following:

- Each node forwards the packets towards the destination using the shortest hop route. A hop-count is associated with each packet, incremented by one once a node forwards it. A packet is discarded if its hop-count exceeds the threshold value.
- If the destination node is one of the neighbor nodes, the packet is forwarded directly. Otherwise it is forwarded to the next-hop node in the shortest path to the destination with temperature less than or equal to the threshold value. The threshold temperature of a node is derived from the average temperature of the neighbor nodes and the own temperature of the node.
- If the temperature of the next-hop in the shortest path to the destination is higher than the summation of the source node's temperature and the threshold temperature, then the node identifies it as a hotspot and bypasses it by forwarding the packet to non-visited neighbor node with least temperature. Each packet maintains a list of the visited nodes to avoid routing loops.

In this scheme, each node calculates the threshold value based on temperature of its neighbor nodes and its own temperature. This makes it different from the other thermal-aware routing protocols where threshold is a predefined value [15].

#### 4.2.6. Routing Algorithm for Network of Homogeneous and ID-Less Bio-Medical Sensor Nodes (RAIN)

RAIN [57] is a routing protocol that aims to reduce the average temperature rise and average power consumption of bio-medical sensor nodes. The three phases of RAIN are: setup phase, routing phase and status update phase. The term ID-less does not mean that the sensor nodes do not use IDs at all. Instead of using static global IDs, RAIN uses temporary IDs. These temporary IDs are randomly generated during the setup phase between one and (2^16^−1), valid for its operation life. ID ‘zero’ is reserved for the sink node. All nodes including the sink node, broadcast their IDs through hello packets.

In the routing phase each generated data packet having a unique packet ID [N,T,R], is routed towards the destination node using the multi-hop technique, where ‘N’ is the node's ID, ‘T’ is the time at which the data packet is generated at the source node, and ‘R’ is a random number. A hop-count is associated with each packet to stop the infinitely routing loops. If the hop-count exceeds the threshold value of HOP_THRESH, then it is discarded. Each node maintains the list of the packets IDs to prevent the duplicate transmission of packets and a data packet is dropped if its ID already exists in that list. Each node also maintains the estimated temperatures of the neighbor nodes by monitoring their communication activities. If destination node is among the neighbor nodes, the packet is forwarded to it; otherwise it is forwarded to the neighbor node using probability which is inversely proportional to its temperature. In status update phase, the sink informs all its neighbor nodes if it receives any data packet by sending the packet's ID to reduce the power consumption of the node.

#### 4.2.7. Thermal-Aware Shortest Hop Routing (TSHR)

Tabandeh *et al.* proposed in [58] a thermal-aware shortest hop routing protocol for *in vivo* bio-medical sensor networks that intends to reduce the temperature rise without affecting the packet delivery delay and power consumption of bio-medical sensor nodes. TSHR is suitable for applications having high priority for packets delivery to the destination. It also considers the retransmission of the dropped packets. The two phases of TSHR protocol are: setup phase and routing phase. In setup phase each node builds its routing table while in routing phase each node route the packets to the destination based on shortest path. TSHR defines two thresholds to control the nodes' temperature: T_s_ and T_Dn_. ‘T_s_’ is fixed and the nodes are not allowed to go beyond it, while ‘T_Dn_’ is dynamic and is based on the node's own temperature as well as on its neighbor nodes' temperature and can be calculated as:

(2) $$T_{DN}={\text{temp}}_{n}+0.25\sqrt{{\text{temp}}_{n}}+0.25\sqrt{{\text{avg}}_{n}}$$

where ‘temp_n_’ is the node's temperature and ‘avg_n_’ is the average temperature of its neighbors. A node is declared as hotspot node with greater temperature as compared to ‘T_DN_’.

#### 4.2.8. A New Energy-Efficient Routing Protocol (M-ATTEMPT)

M-ATTEMPT [59] is an energy efficient and thermal aware routing protocol for WBSNs to reduce the nodes' temperature as well as to decrease the delay for the critical data using heterogeneous bio-medical sensor nodes. In the network architecture of this scheme, the sink node (base station) is placed at the center while nodes with high data rates are placed at less mobile places of the human body. For critical or query driven data packets, the sensor nodes increase their transmission power to forward the data packets directly to the sink node (single-hop) while for ordinary data packets multi-hop communication is used. The sensors having ordinary data packets cannot forward them until the sink received all the critical or query driven data packet. In case of multi-hop communication, a route with less hop-count is selected if two or more routes are available. If two or more next-hop neighbor nodes have the same hop-count then the neighbor node with less energy consumption to the sink is selected. To control the rise in temperature, M-ATTEMPT defines a threshold and if any node's temperature goes beyond that threshold, it breaks all the routes with the neighbor node. However, if the node's temperature reaches to the threshold after receiving a data packet, it resends that packet to previous node and the previous node marks it as a hotspot.

The four different phases of M-ATTEMPT are: initialization phase, routing phase, scheduling phase, and data transmission phase. In initialization phase all nodes broadcast the hello packet, while in routing phase routes with less hop-counts are selected within the available routes based on aforementioned procedure. The sink node creates a Time Division Multiple Access (TDMA) schedule for all root nodes in the scheduling phase, while the root nodes send their data to the sink node during the data transmission phase.

#### 4.2.9. Comparative Study of Temperature-Aware Routing Protocols

All the aforementioned temperature-aware routing protocols ultimately intend to reduce the temperature rise of implanted bio-medical sensor nodes. A comparison in terms of temperature rise, packet discarding mechanism, addresses scheme, delay, packet delivery ratio and energy consumption with other state-of-the-art schemes is given in Table 2.

The first routing protocol that addresses the overheating issue of implanted bio-medical sensor nodes in WBSNs is TARA [28]. It performs better in terms of reducing the temperature rise, and load balancing as compared to SHR, but its withdrawal strategy increases the number of transmissions, energy consumption, and end-to-end delay. The delay issue due to withdrawal strategy in TARA is addressed in LTR [55] and ALTR [55]. As compared to TARA, both LTR and ALTR exhibit robustness at different packets arrival rates, and perform well in terms of temperature rise, energy consumption, packet delivery ratio, and end-to-end delay. Compared to LTR, ALTR incurs low end-to-end delay and high packet delivery ratio. The disadvantages of LTR and ALRT are that it is not guaranteed that the packets are transmitted in the correct direction towards the destination, as they are only looking at temperature rise of bio-medical sensor nodes. Furthermore, in ALTR, if the hop count exceeds the threshold value, the packets might be transmitted through hotspots, resulting in temperature rises. To address these disadvantages of LTR and ALTR, LTRT [56] focuses on end-to-end route temperature and this result in less temperature rise and delay as compared to LTR and ALTR.

To control the temperature rise, HPR [29] prevents the formation of hotspots by sending the data packets to the coolest nodes. Furthermore, the threshold temperature of any sensor node is computed based on temperature of its own along with its neighbors, which makes it different from other temperature aware routing protocols [15]. As compared to TARA [28], HPR performs better to reduce temperature rise, delay and packet loss ratio. The authors of TSHR have modified the HPR [29] by introducing a fixed threshold along with the dynamic threshold. TSHR [57] performs well in terms of node's temperature rise, the packet delivery delay (especially at high packet arrival rates) and packet loss ratio then TARA [28] and LTR [55]. While as compared to HPR, TSHR has lower packet drop ratio, higher packet delivery delay and almost same temperature rise. TSHR along with TARA and ALTR have no packet drop mechanism.

By comparing TARA [28], LTR [55], ALTR [55], LTRT [56], HPR [29] and TSHR [57] in terms energy consumption, packet delivery delay, packet delivery ratio and temperature rise, we conclude the following: LTRT [56] outperforms the others in terms of energy consumption while TARA [28] is the worst. To reduce the packet delivery delay, HPR [29] performs better as compared to others, and due to the withdrawal strategy of TARA [28], it has the highest packet delivery delay. In terms of packet delivery ratio, TARA [28] drops more packets due to longer paths while TSHR [57] reduces the packets drop by adopting the shortest paths. Finally, to reduce the temperature rise, HPR [29] results in less temperature rise as compared to others.

All temperature aware routing protocols use a global address scheme except RAIN [58], which uses a randomly generated local address scheme. As compared to C-FLOOD, RAIN performs well to reduce temperature rise, power consumption, and packet delivery delay, but has slightly lower packet delivery ratio as compared to C-FLOOD. Finally, the last temperature aware routing protocol M-ATTEMP [59] has increased the packet delivery ratio and decreased the energy consumption and temperature rise as compared to multi-hop communication.

### 4.3. Cluster-Based Routing Protocols

The third class of WBSNs routing protocols is cluster-based routing protocols, where the bio-medical sensor nodes or sensing nodes are divided into one or more clusters. Different methods are used to select one of nodes of the specific cluster, as a Cluster Head (CH) and the data is send to the sink (base station) through these CHs to reduce the direct communication between the sensing nodes and the sink. The following sections give the overview of some of the important cluster-based routing protocols for WBSNs.

#### 4.3.1. Hybrid Indirect Transmission (HIT)

In [60] the authors proposed a data gathering protocol named Hybrid Indirect Transmission (HIT) based on hybrid architecture of one or more clusters where each cluster is capable of multiple multi-hop transmissions. HIT uses parallel processing both in intra-cluster and inter-cluster communication to minimize the energy consumption as well as the network delay. The details of the HIT procedure are as follows: initially, one or more cluster heads are elected and then these cluster heads broadcast their status throughout the network to form one or more clusters. After cluster formation, upstream and downstream relationships for each cluster are formed due to which multiple routes are figured out within each cluster from the sensor nodes to the cluster heads. Next each node finds its blocking set which is a list of nodes not allowed to communicate at the same time with that node. Finally, after calculating the Time Division Multiple Access (TDMA) schedule, the sensor nodes transmit the sensed data to the cluster head through upstream neighbors.

#### 4.3.2. AnyBody

AnyBody [61] is a cluster-based self organizing data gathering protocol, designed to reduce the direct transmission of the sensor nodes with remote based stations. AnyBody uses LEACH given in [62]—which selects the cluster head at regular time intervals to balance the energy consumption and cluster head aggregates the data and sends to the remote station. In LEACH it is assumed that all nodes are within the range of remote base station. AnyBody addresses this problem by using a density-based cluster head selection method and using these cluster heads to build a backbone network. The five steps of AnyBody are: neighbor discovery, density calculation, constructing cluster head, setting up the backbone, and setting up the routing path.

**Figure 12.**
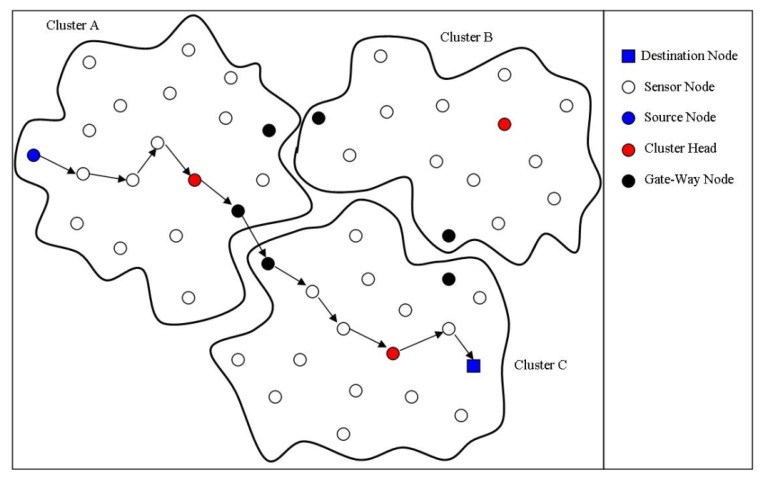
Hierarchical structure of cluster-based routing.

In the first step, the one-hop neighbors are discovered by sending a Hello1 message during the first time frame and two-hop neighbors by sending a Hello2 message during the second time frame. In the second step, each node calculates its density based on its two-hop neighborhood information and shares the density information with its neighbor nodes by broadcasting a Hello3 message. In the third step, each node sends the list of its one-hop neighbor nodes to the neighbor node with highest density using a Join message and if it has the highest density then it will not send any messages. The cluster head is selecting based on the highest local density and nodes are grouped in clusters. In the fourth step, each cluster head figures out the gateway nodes. The gateway nodes are the nodes having neighbors outside its cluster. They are used to forward the messages from the gateway nodes of other clusters to its cluster head and forward the messages of its cluster head for other clusters to their corresponding gateway nodes. In the last step, the sink sends a gradient_setup(1) message to its all directed connected cluster heads, then these cluster heads send gradient_setup(2) messages to other cluster heads directly connected with them and this process continues until all cluster heads construct their routing paths. Each cluster head sends the gradient setup messages to all directed connected cluster heads except of the one from it has received. Figure 12 show the hierarchical structure of cluster-based routing.

#### 4.3.3. Comparative Study of Cluster-Based Routing Protocols

Limited energy is the main constraint in WBSNs as well as in other WSN applications. In WSNs, efficient cluster mechanisms are used to reduce the number of direct communications of the sensor nodes with the base station, to decrease the power consumption, enhance the link quality and thus increasing the lifetime of the network [62]. The comparison of both cluster-based routing protocols for WBSNs with other state-of-the-art schemes is summarized in Table 3, in terms of delay, packet delivery ratio, security and energy consumption.

HIT [60] performs better in reducing the energy consumption as compared to LEACH [63], PEGASIS [64] and direct transmission for small number of nodes, however it consumes more energy in dense networks. Furthermore, HIT also reduced the network delay to gather the data. The main advantage of AnyBody [61] is that the number of clusters remains almost constant by increasing number of nodes, while in LEACH with the increase in nodes results increase in number of clusters (5% of the nodes). This results in a bigger cluster size of AnyBody as compared to LEACH. Furthermore, AnyBody also reduces the cost of setting up the cluster.

HIT [60] results in low packet delivery delay and energy consumption and does not consider packet delivery ratio. On the other hand, AnyBody [61] improves the packet delivery ratio and does not consider the packet delivery latency and energy consumption.

### 4.4. Postural-Movement-Based Routing Protocols

The network topology of WBSNs or the link between two nodes often faces the problem of partitioning or disconnection because of the body postural movements. In this class of WBSNs routing protocols, different researchers have tried to solve the problem of link disconnection by defining a cost function, which is updated periodically. These protocols choose routes from the bio-medical sensor nodes to the base station with minimum cost to forward the data packets. Some of these routing protocols are discussed in the following sections.

#### 4.4.1. On-Body Store and Flood Routing (OBSFR)

**Figure 13.**
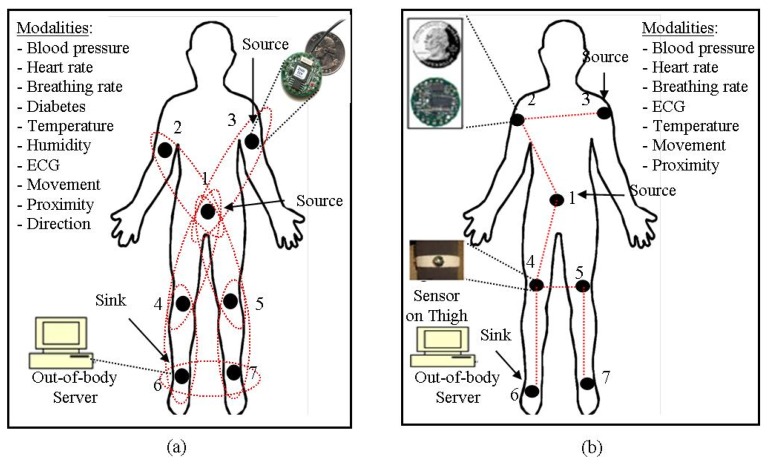
Network Architecture of (**a**) OBSFR [21] (**b**) PRPLC [22].

In [21], the authors presented the location based store-and-flood routing protocol (OBSFR), that in the presence of frequent partitioning intends to reduce the end-to-end delay and hop-count. OBSFR is a modified flooding protocol designed for partitioned networks. In the flooding mechanism, a source node sends multiple copies of the data packets towards the destination (sink node) through multiple routes. The network architecture used in this scheme is consists of seven sensor nodes: two at upper arms, two at thighs, two at ankles and one in the waist area, as shown in Figure 13a, redrawn from [21]. Thus forming a mesh topology divided in one or multiple simultaneous network partitions. The sensor node attached at the right ankle is assigned as a sink node (base station). The sink node is responsible for collecting the raw data from the other bio-medical sensor nodes and forward the processed results to the server located outside of the body, resulting in multi-point-to-point routing.

In OBSFR, each packet carries a list of the node-IDs showing its path from the source node along with a unique identifier (source_ID, seq_No.). When a node receives a packet, it stores the packet in its buffer, and starts search for the nodes whose IDs are not listed in the received packet's IDs list. Once found one or more such nodes then it forwards the packets using a broadcast mechanism.

#### 4.4.2. Probabilistic Routing (PRPLC)

In [22] Quwaider and Biswas proposed a store-and-forward packet routing protocol based on postural partitioning to minimize end-to-end packets delay by dynamically choosing routes having low storage/buffering delays. As shown in Figure 13b, redrawn from [22], the network architecture used in this scheme consists of seven bio-medical sensor nodes: two on the upper arms, two on the thighs, two on the ankles and one on the waist area, forming a mesh topology divided into one or multiple simultaneous network partitions. The sensor node attached on the right ankle is assigned as a sink node (base station). The sink node is responsible for collecting the raw data from the other bio-medical sensor nodes and forward the processed results to the server located outside of the body, resulting in multi-point-to-point routing.

The impact of human posture mobility on the network partitioning was observed by asking the human test subjects to follow a predefined sequence of postures. In this protocol, the likelihood of any link ‘*L_i, j_*’ between any two nodes ‘*i*’ and ‘*j*’ to be connected for a discrete time slot ‘*t*’ is defined as Link Likelihood Factor (LLF) denoted by $P_{i,j}^{t}(0\leq P_{i,j}^{t}\leq 1)$. The LLF is supposed to be updated dynamically at regular interval ‘*t*’ as:

(3) $$P_{i,j}^{t}=\{\begin{array}{ll} P_{i,j}^{t-1}+\left(1-P_{i,j}^{t-1}\right)\omega & If\_\text{Link}\_L_{i,j}\_is\_\text{Connected}\\ P_{i,j}^{t-1}.\omega & If\_\text{Link}\_L_{i,j}\_is\_\text{Disconnected} \end{array}$$

where ‘*ω*’ is a constant and its range is 0 ≤ *ω* ≤ 1. For a low value of ‘*ω*’, LLF increases slowly (connected link) and decreases fast (disconnected link) identifying historically bad links. While for a high value of ‘*ω*’, it increases fast (connected link) and decreases slowly (disconnected link) identifying historically good links. The term ‘*ω*’ should be able to know the long-term history knowledge of the link based on the Historical Connectivity Quality (HCQ) discussed in [65]. All the nodes share their LLF towards their neighbors as well as towards the sink node with their neighbors via hello messages all the time. Every node will forward a packet to its neighbor only if its own LLF towards the sink node is less than or equal to that neighbors' LLF towards the destination. Otherwise, it stores the packet in its buffer and waits for an appropriate next-hop node.

#### 4.4.3. DTN Routing with Dynamic Postural Partitioning (DVRPLC)

DVRPLC [23], present a store-and-forward packet routing protocol based on postural partitioning, aiming to minimize end-to-end packet delays by choosing routes with high likelihood. The routing cost of a link ‘*L_i,j_*’ between any two nodes ‘*i*’ and ‘*j*’ in a discrete time slot ‘*t*’ is referred as Link Cost Factor (LCF) denoted by $C_{i,j}^{t}\left(0\leq C_{i,j}^{t}\leq C_{\max}\right)$, which updates dynamically after the ‘*t^th^*’ time slot as:

(4) $$C_{i,j}^{t}=\{\begin{array}{ll} C_{i,j}^{t-1}\left(1-\omega_{i,j}^{t}\right) & If\_\text{Link}\_L_{i,j}\_is\_\text{Connected}\\ \omega_{i,j}^{t-1}(C_{i,j}^{t-1}-1)+1 & If\_\text{Link}\_L_{i,j}\_is\_\text{Disconnected} \end{array}$$

The constant $\omega_{i,j}^{t}\left(0\leq \omega_{i,j}^{t}\leq 1\right)$ is based on Historical Connectivity Quality (HCQ) discussed in [65]. DVRPLC uses the same routing procedure as that used by PRPLC [22] with a difference of selecting links with lower costs to minimize the end-to-end cumulative cost, which outperforms the PRPLC [22].

#### 4.4.4. Opportunistic Routing

In [20], the authors have proposed an opportunistic routing protocol by considering the moving nature of the human body. The network model used in this protocol is very simple, where a bio-medical sensor node on the chest senses the data and sends it to the sink node on the wrist. Moreover, there is a relay node on the wrist to facilitate the communication between the bio-medical sensor node and the sink node. During human body movements (walking or running) the wrist (having the sink and relay nodes) moves forward and backward resulting into two types of possible communication: None Line of Sight (NLoS) communication—when the wrist is at the back of the body and Line of Sight (LoS) communication—when the wrist is at the front of the body. Both NLoS and LoS communications are considered to have the same probability, *i.e.*, 0.5.

The sensor node sends Request To Send (RTS) packet, if it has data packets to send to sink node, which will only be received by LoS nodes. If the RTS packet is acknowledged within a specific time interval, *i.e.*, the sink node is in LoS with sensor node, then the sensor node starts forwarding the data packets directly to the Sink. On the other hand, if the RTS packet is not acknowledged within specific time of interval, *i.e.*, the sink node is in NLoS with the sensor node, then the sensor node will send a wakeup packet to relay node. Once the relay node is ready for communication, it will inform both the sensor node and sink node to start communication. After successfully receiving all the data packets, the sink node will send a Receive Acknowledge (RAck) packet to the sensor node. If the sensor node does not receive the RAck packet, then the aforementioned procedure will be repeated until successful communication.

#### 4.4.5. Energy Efficient Thermal and Power Aware Routing (ETPA)

In [66] Abolhasan and Lipman proposed an energy efficient, thermal and power aware routing protocol, based on the posture-movement of the human body. ETPA aims to decrease the temperature rise and power consumption. ETPA uses the same network architecture used by PRPLC [22]. In this protocol the frames are divided into time slots using a Time Division Multiple Access (TDMA) scheme, where each node transmits in its own time slot. During each cycle (after every four frames), all nodes broadcast their hello messages to their neighbors containing the temperature and residual energy and then every node estimates the received power from neighbors nodes. ETPA uses temperature, residual energy and transmission power of the node to calculate the cost function. When a node has a packet to send, it searches for an efficient route with minimum cost. If it finds nodes with an efficient route, it forwards the packet, otherwise it buffers the packet. A packet is dropped if it has been buffered for more than two frames. In order to decrease the delay, each packet can only pass through a pre-defined number of hops (max_hop_count), otherwise it will be dropped.

#### 4.4.6. Exploiting Prediction to Enable Secure and Reliable Routing (PSR)

In [67] the authors present a distributed prediction based secure and reliable routing framework, which can be integrated with any other routing protocol to achieve improvement in its reliability and security performance. A backbone link is a communication link between any two neighbor nodes which are at a constant distance from each other. These backbone links form a shortest path tree at the sink node. PSR selects next-hop node with highest predicted link quality based on the previous link quality measurements. PSR uses lightweight approaches for source and data authentications for secure communication from the three types of data injections attacks: exhaustive source authentication attacks (false authentication requests), exhaustive data authentication attacks (false date packets), and data replay attacks (eavesdropped security materials to introduce fake data packets).

PSR is categorized into two prediction based sub-algorithms: next hop selection algorithm and data transmission algorithm. In the next hop selection algorithm, each node maintains a matrix to store the link quality measurements for previous pre-defined time slots. The link quality matrix is used to build the prediction model to predict the best link quality. In absence of the prediction model, the data packets will be forwarded using the backbone link based shortest path tree. While in the data transmission algorithm first the source authentication will take place and then the data authentication. The source authentication consists of two steps. In first step, a node will broadcast a source authentication request containing an identity based signature using the signature scheme discussed in [68]. Each of the neighbor nodes will verify the signature and if equality holds then it will reply in the second step, otherwise the authentication process is stopped. The data authentication process will be initiated where a set of secret tokens will be shared among any pair of nodes after they successfully authenticate each other. For every data packet received by the neighbor node is checked for a valid token to authenticate the packet. In the absence of a valid token, the source authentication will start. To avoid any occasional transmission failure, each node initiates the source authenticates for the maximum number of time.

#### 4.4.7. Comparative Study of Postural-Movement-Based Routing Protocols

Due to the movements of human body, the links among the sensor nodes often face disconnection problems. Different researchers have tried to solve this problem, which are discussed above. In Table 4 the aforementioned postural movement based routing protocols for WBSNs are compared with other state-of-the-art schemes in terms of delay, packet delivery ratio and energy consumption.

OBSFR [21] has increased the pocket delivery ratio and decreased the average delay as compared to the PROPHET [69] and Relative LOCation based Forwarding (RLOCF), but still there is a unique packet loss due to the partition packet saturation. However, OBSFR [21] has high energy consumption and can only be applied to networks with a few nodes due to the list of IDs in each packet. The performance of PRPLC [22] is better in terms of packet delivery ratio and end-to-end delay as compared to generic probabilistic routing protocol PROPHET [69] but still it has high end-to-end packet delay and low packet delivery ratio as compared to the on-body store and flood routing (OBSFR) [21]. DVRPLC [23] reduces the average delay and enhances the packet delivery ratio as compared to PRPLC [22] and PROPHET [69]. However, OBSFR [21] performs better in terms of average delay and packet delivery ratio as compared DVRPLC [23]. Furthermore, DVRPLC having high packet hop count resulting in longer routes as compared to PRPLC [22].

The opportunistic protocol [20] has half of the energy consumption for the relay node, and same energy consumption for the sensor node as compared to that multi-hop communication as discussed in [70], *i.e.*, the overall energy consumption is in between the single-hop and multi-hop communication. However, the individual energy level of the sensor node is not considered. Furthermore, with the increase in the number of sensor nodes will increase the traffic load on the relay node. As compared to PRPLC [22], ETPA [66] shows an efficient performance in terms of the temperature rise, while packet delivery ratio and packet delay almost remain the same. However, the average packet hop is slightly higher than PRPLC. PSR [67] framework is suitable for wireless body sensor networks having a few number of sensor nodes because of the huge overhead of the link quality matrix.

By comparing the postural-movement-based routing protocols in terms of packet delivery delay, packet delivery ratio and energy efficiency, we conclude the following: among the aforementioned postural-movement-based routing protocols for WBSNs, OBSFR [21] performs better to reduce the packet delivery delay. In terms of packet delivery ratio, ETPA [66] achieves highest packet delivery ratio compared to others. Finally, opportunistic postural movement based routing protocol [20] has lowest energy consumption among all.

### 4.5. Cross-Layered Routing Protocols

The last category of the WBSNs routing protocols discussed in this paper is the cross-layered routing protocols. These protocols address and try to solve the issue and challenges of both network and MAC layers at the same time to improve the overall performance of the network. We have discussed some of the important cross-layered routing protocols in the following sections.

#### 4.5.1. Wireless Autonomous Spanning Tree Protocol (WASP)

**Figure 14.**
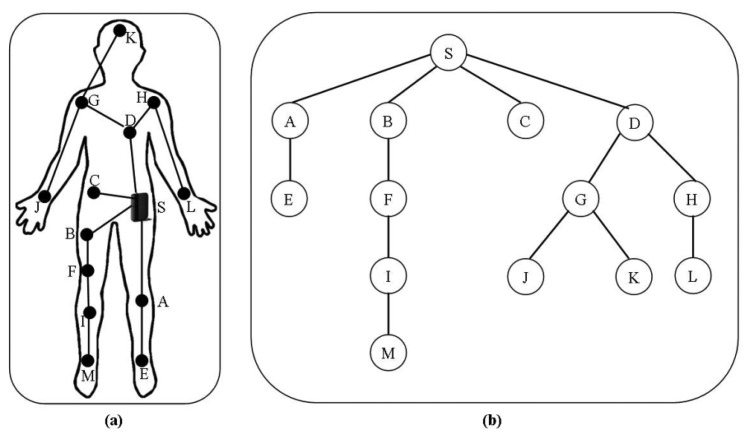
(**a**) Network on the body (**b**) Abstract view of the network [71].

In [71] the authors proposed a cross-layered protocol, named as wireless autonomous spanning tree protocol. WASP divides the time axis into slots in distributed manner, known as WASP-Cycles. The spanning tree is set up automatically as shown in Figure 14, redrawn from [71]. This spanning tree is used to send the data to the sink, and incorporates the medium access control and routing.

Each node sends a WASP-scheme to its children (nodes one level lower in the spanning tree) to inform them about the slot they can use to send the data. The children nodes will reply to their parent nodes (nodes one level up in the spanning tree) through the same WASP-scheme. WASP-scheme is unique for every node and is generated at the source node. Through these schemes the parent node controls the traffic of its children nodes and at the same time the children nodes can use it to ask their parent nodes for more resources. As the WASP-scheme is generated at the source node and used by both parent and children nodes resulting in minimum coordination overhead.

#### 4.5.2. Cascading Information Retrieval by Controlling Access with Dynamic Slot Assignment (CICADA)

CICADA proposed in [72], is a cross-layered low energy protocol, designed for Time Division Multiple Access (TDMA) scheduling based on multi-hop mobile body area networks. This protocol builds a spanning tree same as WASP [71], as shown in Figure 14, redrawn from [71], and slots of the time are assigned in distributed manner and the known length of each cycle makes the slot synchronization possible. The parent nodes are responsible for telling their children nodes when they can communicate. CICADA is the improved version of WASP discussed in [71].

This scheme divides each cycle into two sub-cycles: the control sub-cycle and the data sub-cycle. Each sub-cycle allocates the slots according to its own scheme: the control scheme and the data scheme, and both are forwarded during the control sub-cycle from the parent nodes to their children nodes. Each control scheme shows the sequence, in which the children nodes can send their control scheme to their parent nodes, the length of control sub-cycle and the depth of the tree. At the end of the control sub-cycle, the data sub-cycle starts. The data scheme consists of length of data period and length of waiting period. During the data period the children nodes send the data packets to their parent nodes while during the waiting period the nodes can go to sleep mode to save the energy. Each parent node constructs a table of its children nodes containing the number of slots they need to transmit the data to their parent node and the number of slots they required to receive the data from their children nodes. Each data sub-cycle has a slot for new nodes to join the tree. Each new child node is allowed to send JOIN-REQUEST message in that slot after hearing to the data scheme of the desired parent node.

#### 4.5.3. Timezone Coordinated Sleeping Scheduling (TICOSS)

In [73] Ruzzelli *et al.*, proposed a cross layer protocol, named as TICOSS, designed to improve the IEEE 802.15.4 MAC layer standard. TICOSS is based on MERLIN discussed in [74], which supports multi-hop communication over IEEE 802.15.4 by dividing the network into time zones. This protocol optimizes the IEEE 802.15.4 in the following three perspectives:

- It provides the nodes to use the alternate periods of activity and inactivity to reduce the energy consumption,
- It reduces the packet collisions occurred due to the hidden terminals, and
- It facilitates the nodes to forward the data packets towards the coordinator (PAN in case of IEEE 802.15.4) using shortest path routing.

In the association phase, TICOSS replaces the 64-bits node ID used in IEEE 802.15.4 by a 16-bits node ID. All nodes associate with the network, after exchanging the required information with the coordinator. After association phase, TICOSS divides and synchronize the network into time zones. First of all the coordinator broadcasts its initial zone message containing its time zone (TxZone, which is zero) and clock information to the neighbor nodes. After adjusting their time zones to ‘TxZone+1’ and internal clock, the neighbor nodes of the coordinator forward a new zone message to their neighbors. This process continues until all nodes set their time zone and update their internal clocks. To support node's mobility, addition or removal, TICOSS uses a predefined expiration time for each time zone, and the time zone of any node expires if it does not receive time zone update message, which all nodes broadcast after a specific interval of time. Each node maintains a table to store the updates containing the timestamps and source node ID, which will help the receiving nodes to figure out the updates of the exiting nodes and discard them. To allocate the time slots to all nodes TICOSS uses V-scheduling table, so that all nodes know their periods of activity and inactivity. Nodes have same time zone communicate in the same time slot. V-table supports three sort of transmission: upstream transmission, downstream transmission and local broadcasts.

#### 4.5.4. Biocomm and Biocomm-D

In [75] a cross layer medium access control (MAC) and routing protocol, named Biocomm, is proposed. This protocol aims to optimize the overall performance of the network in terms of avoiding hotspot formation, saving the energy of implanted bio-medical sensor nodes, and at the same time reducing the network traffic.

The interaction of the MAC and network layers to optimize the overall performance of network is supported by Cross-layer Messaging Interface (CMI), which helps them to exchange their status information with each other. Both MAC and network layers keep a record of the status of the neighbor nodes in a Neighbor Status Table. MAC logic sets the Free (F) or Blocked (B) status of the neighbor nodes and the MAC layer informs the network layer about any change in the status of neighbor nodes by sending FREE or BLOCKED message through CMI to update the Neighbor Status Table of the network layer. Similarly, the network layer keeps the MAC layer informed about any free spaces in its packet buffer through CMI and allows the MAC layer for higher frame transmission. Each node tries to use the shortest path in order to send the data packets to the destination node, unless and until it finds a hotspot and/or sleeping node in the path. To prevent infinite routing loops, a hop-count is associated with each packet and if the hop-count for any packet exceeds a predefined threshold, then that packet is dropped. The modified version of Biocomm is Biocomm-D, which intends to handle delay-sensitive data packets.

#### 4.5.5. Comparative Study of Cross-Layered Routing Protocols

The Cross-layered approach enhances the coordination between two or more layers without affecting the original functionalities of the layers. It has attracted the interest of researchers and engineers due to its effectiveness in WSNs. Table 5 provides the comparison among different cross-layered protocols for WBSNs in terms of delay, packet delivery ratio and energy consumption.

WASP [71] increases the packet delivery ratio and decreases the energy consumption as well as end-to-end delay. However, it does not consider the link quality, and does not support two-way communication. Another cross-layered routing protocol for WBSNs is CICADA [72], which is the improved version of WASP [71] and has reduced the energy consumption of sensor nodes by having enough sleep time as compared WASP [71]. TICOSS [73] is a cross-layer protocol designed to improve IEEE 802.15.4 and it performs better in terms of energy consumption, packet delivery ratio, and packet delivery delay. Both Biocomm [75] and Biocomm-D [75] perform better in terms of temperature rise, network throughput, energy consumption of nodes, packet loss percentage due to Time-To-Leave (TTL) expiration and packet loss percentage due to buffer overflow, as compared to HPR [29], but Biocomm-D [75] has slightly high temperature rise as compared to Biocomm. Furthermore, Biocomm [75] has a high average packet delivery delay as compared Biocomm-D [75], HPR [29], while Biocomm-D [75] has low average packet delivery delay as compared HPR [29].

By comparing the cross-layered routing protocols for WBSNs in terms of energy efficiency, packet delivery ratio and average delay, we conclude that CICADA [72] and TICOSS [74] consume less energy as compared to other state-of-the-art schemes. In terms of packet delivery ratio, WASP [71] performs better as compared to others, while to reduce the packet delivery delay, CICADA [72] outperforms other state-of-the-art approaches.

## 5. Conclusions

WBSN is a technology that can provide a paradigm shift towards proactive management by focusing on prevention and early detection of different diseases. It can revolutionize the next generation healthcare issues and reduce healthcare costs. Designing routing protocols for WBSNs is a challenging task due to unique in-body and on-body constraints. In this paper, first we discussed the architecture of Wireless Body Sensor Networks (WBSNs) and then the routing issues and challenges of WBSNs. We have provided a comprehensive review of existing routing protocols specifically designed for WBSNs. Based on their nature and structure, the routing protocols have been classified as QoS-aware routing protocols, temperature-aware routing protocols, cluster-based routing protocols, postural-movement-based routing protocols and cross-layered routing protocols. We have critically analyzed each routing protocol by comparing its relative performance against other state-of-the-art schemes and have identified the relative strengths and weaknesses of each routing protocol.

Different routing challenges in WBSNs are being considered in different categories of routing protocols, but still a lot of work needs to be done. The proposed QoS-aware routing protocols consider the nature of vital sign-related data of patients and other required quality of service parameters but they do not consider the postural-movements of the human body or the thermal effects of implanted bio-medical sensor nodes. The routing protocols that consider the postural-movements of the human body don't consider the temperature rise issue and data-centric quality of service issues. While the routing protocols that considers the temperature rise issue due to antenna radiation absorption and power consumption of sensor node circuitry but they do not take care of other routing issues.

The future routing protocols for WBSNs should provide energy efficient and reliable communication among heterogeneous types of bio-medical sensor nodes in real-time applications. They should also take care of latency, reliability, mobility, thermal-effects and energy consumption. Similarly, more accurate and efficient network architectures should be developed for better routing in WBSNs.

## Figures and Tables

**Table 1.** QoS-aware routing protocols.

**Protocol**	**Characteristics**						

	**Goal**	**Network Size**	**Network Throughput**	**Mobility**	**Delay**	**PDR**	**Energy Cons.**
Routing Service FW (2007)	To provide priority based routing & user specific QoS Support	Small	N/A	Yes	N/A	Medium	N/A
RL-QRP (2008)	To achieve high packet delivery ratio and low end-to-end delay	Small	Low	Yes	High	High	N/A
LOCALMOR (2009)	To provide QoS support based on nature of Data	Medium	N/A	Yes	Low	High	High
DMQoS (2011)	To provide Data Centric QoS support	Large	Very Low	Yes	Low	High	Medium
EPR (2012)	To reduce network traffic and energy consumption	Very Small	High	Yes	N/A	High	Low
QPRD (2012)	To reduce end-to-end delay	Very Small	High	Yes	Very Low	High	Low
QPRR (2013)	To enhance end-to-end reliability	Small	High	Yes	N/A	High	Low

**Table 2.** Temperature-aware routing protocols.

**Protocol**	**Characteristics**						

	**Goal**	**Temp. Rise**	**Discarding Mechanism**	**Address Scheme**	**Delay**	**PDR**	**Energy Cons.**
TARA (2005)	To reduce the possibility of overheating	Very High	No	Global	Very High	Very Low	Very High
LTR (2006)	To reduce the temp rise and energy consumption	High	Yes	Global	High	Low	High
ALTR (2006)	To reduce the temp rise, energy consumption and end-to-end delay	Low	No	Global	Medium	High	High
LTRT (2007)	To find routes with minimum temperature	Very Low	Yes	Global	Low	Very High	Low
HPR (2008)	To prevent the formation of hotspots and reduce end-to-end average delay	Very Low	Yes	Global	Low	High	High
RAIN (2008)	To reduce average temp rise and average delay	Very Low	Yes	Local	Low	High	Low
TSHR (2009)	To reduce temp rise, energy consumption and delay	Very Low	No	Global	Medium	Very High	Low
M-ATTEMPT (2013)	To reduce temp rise, energy consumption and delay	Low	Yes	Global	Low	High	Low

**Table 3.** Cluster-based routing protocols.

**Protocol**	**Characteristics**					

	**Goal**	**Metrics**	**Avg. Delay**	**PDR**	**Security**	**Energy Cons.**
HIT (2004)	To maximize the network life and reduce the direct transmissions to the sink	Geographic information & residual energy of the nodes	Low	N/A	Yes	Low
AnyBody (2007)	To reduce the direct transmission of the nodes to the sink node	Nodes density	N/A	Very High	No	N/A

**Table 4.** Postural-movement-based routing protocols.

**Protocol**	**Characteristics**					

	**Goal**	**Mobility**	**Metrics**	**Avg. Delay**	**PDR**	**Energy Cons.**
OBSFR (2009)	To decrease end-to-end delay and hop-count	Yes	Link Quality	1.88 s	83–92%	High
PRPLC (2009)	To reduce end-to-end delay	Yes	Link Quality	3.19 s	82–88%	Low
DVRPLC (2010)	To reduce end-to-end delay	Yes	Link Quality	2.38 s	81–89%	Low
Opportunistic (2011)	To increase the life time of the network	Yes	LoS & NLoS Comm.	N/A	N/A	Very Low
ETPA (2012)	To reduce the temperature rise & energy consumption	Yes	Link quality & nodes temp.	High	Up to 95%	Low
PSR (2012)	To provide reliability and security	Yes	Past link quality measurements	High	Up to 80%	High

**Table 5.** Cross-layered routing protocols.

**Protocol**	**Characteristics**					

	**Goal**	**Mobility**	**Metrics**	**Avg. Delay**	**PDR**	**Energy Cons.**
WASP (2006)	To decrease PLR, energy consumption, & end-to-end delay	No	Hop count and routing	324 milli-seconds	100%	Medium
CICADA (2007)	To reduce end-to-end delay and energy consumption	Yes	Hop count and routing	<0.3 milli-seconds	N/A	Low
TICOSS (2007)	To improve IEEE 802.15.4 standard	Yes	Hop count and routing	N/A	Over 92%	Low
Biocomm & Biocomm-D (2009)	To optimize the overall performance of the network	No	Node temp, hop count, and delay	Medium	75–100%	Medium
